# Gut microbiota and Sjögren’s syndrome: a two-sample Mendelian randomization study

**DOI:** 10.3389/fimmu.2023.1187906

**Published:** 2023-06-13

**Authors:** Yu Cao, Hao Lu, Wangzi Xu, Ming Zhong

**Affiliations:** ^1^ School of Medicine, Xiamen University, Xiamen, China; ^2^ Department of Oral Histopathology, School and Hospital of Stomatology, China Medical University, Liaoning Province Key Laboratory of Oral Disease, Shenyang, Liaoning, China

**Keywords:** Sjoegren’s syndrome, gut microbiota, Mendelian randomization, causal effect, autoimmune disease

## Abstract

**Background:**

The link between the gut microbiota (GM) and Sjögren’s Syndrome (SS) is well-established and apparent. Whether GM is causally associated with SS is uncertain.

**Methods:**

The MiBioGen consortium’s biggest available genome-wide association study (GWAS) meta-analysis (n=13,266) was used as the basis for a two-sample Mendelian randomization study (TSMR). The causal relationship between GM and SS was investigated using the inverse variance weighted, MR-Egger, weighted median, weighted model, MR-PRESSO, and simple model methods. In order to measure the heterogeneity of instrumental variables (IVs), Cochran’s Q statistics were utilized.

**Results:**

The results showed that genus Fusicatenibacter (odds ratio (OR) = 1.418, 95% confidence interval (CI), 1.072–1.874, P = 0.0143) and genus Ruminiclostridium9 (OR = 1.677, 95% CI, 1.050–2.678, P = 0.0306) were positively correlated with the risk of SS and family Porphyromonadaceae (OR = 0.651, 95% CI, 0.427–0.994, P = 0.0466), genus Subdoligranulum (OR = 0.685, 95% CI, 0.497–0.945, P = 0.0211), genus Butyricicoccus (OR = 0.674, 95% CI, 0.470–0.967, P = 0.0319) and genus Lachnospiraceae (OR = 0.750, 95% CI, 0.585–0.961, P = 0.0229) were negatively correlated with SS risk using the inverse variance weighted (IVW) technique. Furthermore, four GM related genes: ARAP3, NMUR1, TEC and SIRPD were significant causally with SS after FDR correction (FDR<0.05).

**Conclusions:**

This study provides evidence for either positive or negative causal effects of GM composition and its related genes on SS risk. We want to provide novel approaches for continued GM and SS-related research and therapy by elucidating the genetic relationship between GM and SS.

## Introduction

The peak incidence of Sjögren’s Syndrome (SS), a chronic autoimmune illness, occurs around the age of 50 (1). Inflammation of the exocrine glands, particularly the salivary and lacrimal glands, is the main side effect of SS and a contributing reason in excessively dry mouth and eyes. Clinical signs of SS might range from those associated with sicca to systemic illness and cancer (2). Formal criteria for the diagnosis are based on the severe immunologic abnormalities of SS, which include the detection of serum anti-Roantibodies antibodies and localized lymphocytic sialadenitis on labial salivary gland biopsy (3). This illness has a heavy impact since there are few viable treatment choices. Thus, it is crucial to investigate the causes of SS in order to aid in the creation of treatment plans that cause little harm or even no adverse effects.

The biggest known symbiotic microbiological communities in the human body is the gut microbiome (GM), which is made up of bacteria, fungi, viruses, and protozoa (4), and comprises 4 trillion microorganisms (5) and 150 000 microbial genomes (6). The development of the human immune system is crucially influenced by the gut microbiota, which also protects against pathogen overgrowth (7). According to a study, the dynamics of human immune cells were connected to the gut microbiome, indicating that the gut microbiome was responsible for the immune system’s regulation (8). Autoimmune illnesses were made more likely by the dysbiosis of the gut microbiome, which impacted immune responses (9). One theory was that autoimmune reactions to nuclear antigens were affected by the presence of commensal GM (10). Previous research has linked the IL-23/IL-17 major cytokine pathway to the development of GM (11) as well as spondyloarthritis (SpA), ankylosing spondylitis (AS), reactive arthritis (ReA) (12), and reactive arthritis induced by bacterial infections. In addition to maintaining intestinal permeability, IL-17 encourages T cell priming and boosts the production of pro-inflammatory cytokines and chemokines by immune cells (13), fibroblasts, endothelium and epithelial cells, and endothelial and epithelial cells (14). The primary source of IL-17 is Th17 cells. Thl7 cell growth results from T cell activation, which is greatly aided by IL-23. Th17 cells contribute significantly to the emergence of SpA by the induction of pro-inflammatory cytokines such IL-17 and TNF-α (15). Moreover, prior research has found that the microbiota diversity of SS is much lower than that of healthy controls (16–22), suggesting that microbial dysbiosis may contribute to the pathogenesis. Nevertheless, whether there is a causal connection between the gut microbiota and SS is yet unknown.

Mendelian randomization (MR) is a unique method to investigate the relationship between GM and SS in this context. To evaluate the causative relationship between exposure and illness outcome, MR constructs IVs of exposure using genetic variations (23). The distribution of genotypes from parent to child is random, therefore common confounding variables have no effect on the connection between genetic variations and outcome, and a causal sequence is justified (24). In this work, two-sample Mendelian randomization (TSMR) study was carried out to assess the causal link between GM and SS using GWAS summary statistics from the MiBioGen and FinnGen consortiums.

## Materials and methods

### The MR study’s assumptions and design

**Figure 1:**
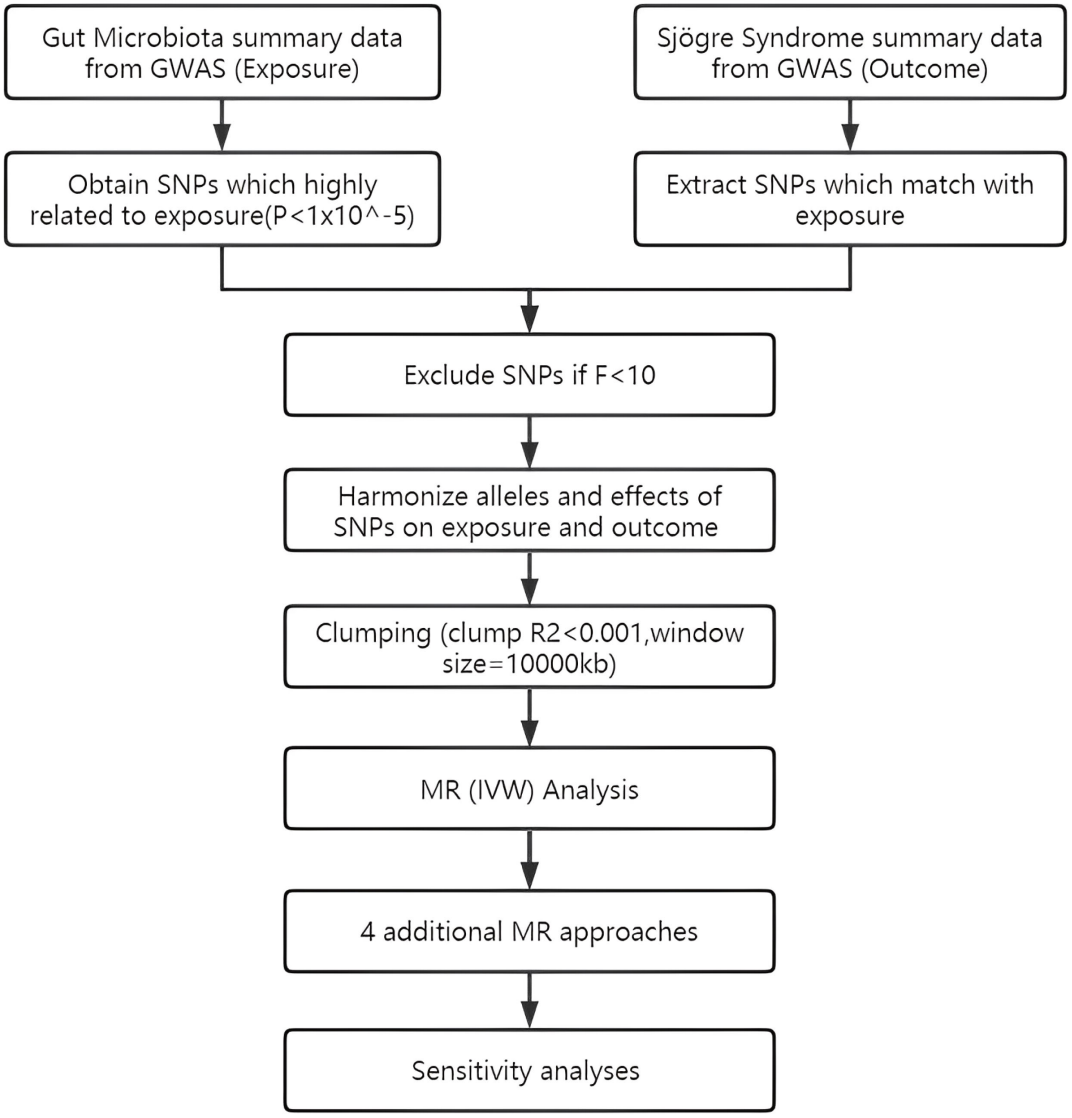
Flowchart of TSMR design.

**Figure 2:**
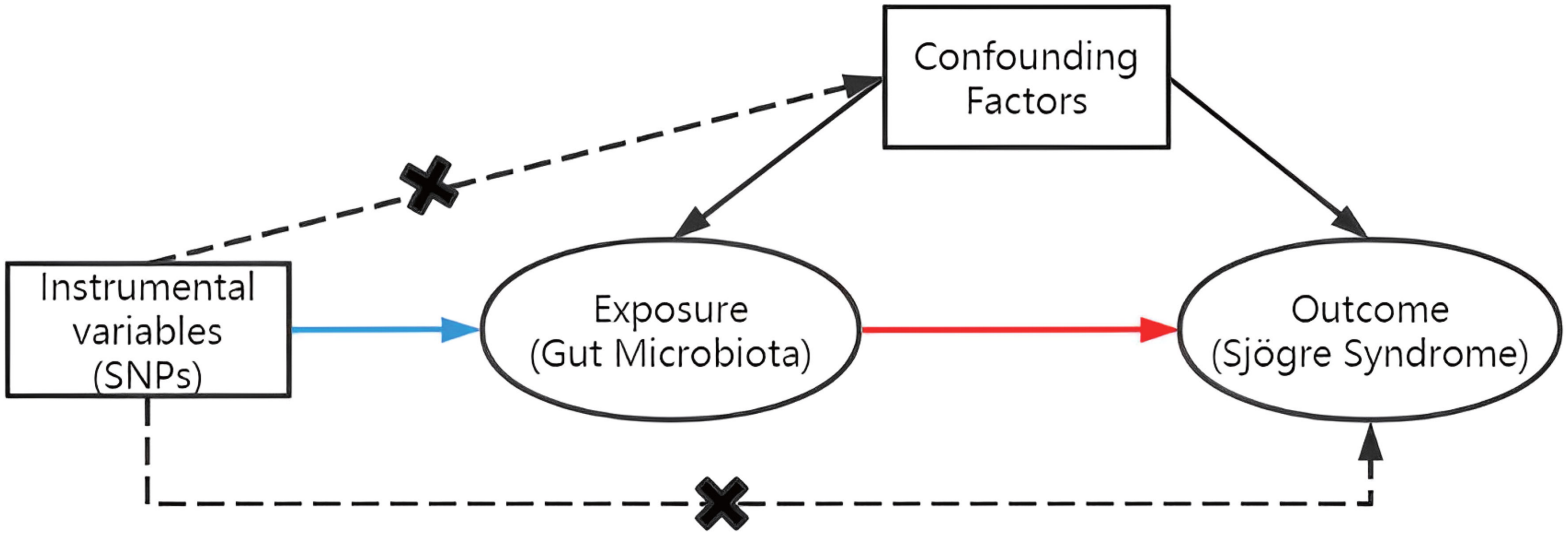
Three assumptions of MR.

To assess the causal connections between GM taxa and SS, TSMR analysis was performed. **Figure 1** shows the flowchart of the MR study between GM taxa and SS. For GM and SS, summary-level data from the genome-wide association studies (GWASs) was acquired. The MR study also met the following 3 assumptions (25) in order to get trustworthy findings (**Figure 2**): (1) The GM taxa must be closely connected to the instrumental variables (IVs) that are ultimately included for usage; (2) There was no interdependence between the included IVs and confounders (affecting GM taxa and SS); (3) There was no horizontal pleiotropy since SS was only impacted by IVs *via* GM taxa. In the meanwhile, our results were published in accordance with the MR-STROBE recommendations (26).

### Data sets

The MiBioGen research is the largest ethnically diverse genome-wide meta-analysis of the GM to date (27). In the GWAS meta-analysis, there were 18,340 people from 24 cohorts. Targeting the V4, V3-V4, and V1-V2 sections of the 16S rRNA gene allowed researchers to characterize the microbial makeup of several cohorts, and 10,000 readings per cohort were selected from all microbiome datasets. An effective sample size of at least 3,000 people and participation in at least three cohorts were the study-wide cutoffs. Through the implementation of a standardized pipeline, microbiome trait loci (mbTL) were mapped to identify genetic loci that affect the relative abundance (mbQTL) or presence (microbiome binary trait loci, or mbBTL) of microbial taxa. 211 bacterial taxa’s worth of accessible GWAS summary statistics were subsequently used into the MR analysis. SS GWAS data containing 2247 cases and 332115 controls is obtained from FinnGen Release 8, published on Dec 1, 2022(https://www.finngen.fi/en) (28). SS patients are defined according to ICD-10 code M35.0, ICD-9 code 7102, or ICD-8 code 73490 (mostly according to ICD-10 code).

The IVs were chosen using the following selection criteria: First, possible IVs were chosen from single nucleotide polymorphisms (SNPs) connected to each species at a significance level of (P = 1x10-5) (29); Second, the linkage disequilibrium (LD) between the SNPs was calculated using data from the 1000 Genomes Project’s European samples as the reference panel, and only SNPs with the lowest P-values were kept among those SNPs with R2 < 0.001 (clumping window size = 10,000 kb); Third, SNPs having a minor allele frequency (MAF) of less than 0.01 were eliminated; Fourth, F-statistic measures the strength of the relationship between genetic variants and exposure, where a higher F-statistic indicates a stronger instrument. The threshold of 10 is widely accepted as it corresponds to an instrument that explains at least 10% of the variance in the exposure variable and has a low probability of weak instrument bias (30). F-statistic for each IV is calculated (F=(beta/se)^2), and only those IVs with an F-statistic greater than 10 were retained.

### Statistical analysis

In this investigation, a variety of techniques were utilized to determine if there was a causal relationship between GM and SS, including inverse variance weighted (IVW), MR-Egger, weighted median, weighted model, MR-PRESSO, and simple model approaches. To evaluate the overall impact of GM on SS, the IVW technique coupled a meta-analysis strategy with the Wald estimates for each SNP. The IVW results would be unbiased if there was no horizontal pleiotropy (31). Based on the premise that instrument strength is independent of direct effect (InSIDE), MR-Egger regression enables the evaluation of pleiotropy using the intercept term. The outcome of the MR-Egger regression is consistent with IVW if the intercept term equals zero, demonstrating the absence of horizontal pleiotropy (32). When up to 50% of IVs are invalid, the weighted median technique enables accurate causal connection assessment (33). The weighted mode estimate has been found to have more power to identify a causal impact, less bias, and lower type I error rates than MR-Egger regression in the event that the InSIDE hypothesis is falsified (33). By eliminating large outliers, the MR-PRESSO analysis finds horizontal pleiotropy and makes an effort to decrease it. Nevertheless, the MR-PRESSO outlier test is dependent on InSIDE assumptions and necessitates that at least 50% of the genetic variations be valid instruments (34). The simple mode approach is also less biased than other methods while being less precise since it can reduce bias (33).

In order to assess the stability of significant results, we carried out additional tests for heterogeneity and horizontal pleiotropy using meta-analytic methods. These tests included the modified Cochran Q statistic and the MR Egger intercept test of deviation from the norm (35). To avoid horizontal pleiotropy brought on by a single SNP, a leave-one-out analysis was carried out, which systematically drops one SNP at a time. The packages “TwoSampleMR” (36) and “MRPRESSO” in R version 4.2.1 were used for every analysis.

### Mapping SNPs to genes

To further understand the mechanism of the influence of gut microbiota on Sjogren syndrome, we entered the SNPs of each Taxa that were significant in the MR analysis as lead SNPs into FUMA GWAS (37) (a platform that can be used to annotate, prioritize, visualize and interpret GWAS results). These SNPs were mapped to genes using the SNP2GENE tool in FUMA. To understand gene interactions at the protein level, PPI networks were constructed for mapped genes using STRING (38) with 0.4 as the recommended minimum interaction index and default values for all other variables. Analysis and display of PPI data using Cytoscape (V3.9.1).

### Deeper MR analysis of transcriptomic

To further verify the causal relationship between mapped genes and SS, we performed a transcriptome Mendelian randomization analysis on them. We obtained cis-eQTLs (cis expression quantitative trait loci, cis-eQTLs) of SNP mapped genes from the eQTLGen consortium (https://eqtlgen.org/). Complete descriptions of the data are accessible in the original publications (39). In a nutshell, the eQTLGen data comprised cis-eQTLs for 16,987 genes and 31,684 blood samples, the majority of which were from healthy people of European ancestry. The whole set of significant cis-eQTL findings (false discovery rate, FDR < 0.05) and allele frequency data was downloaded at 2023/03/10. Using a very low correlation criterion in the setting of a cis-region MR may lead to the loss of causative variants; hence, these eQTLs were clumped using a pairwise linkage disequilibrium (LD) threshold of r2 < 0. 1 (40).The final IVs were obtained for 237 genes from a total of 324 genes. The process of Mendelian randomization was the same as before, but considering the multiple testing problem, we performed an FDR correction, and the result of FDR < 0.05 was considered significant.

## Results

### Causal effects of gut microbiome on SS

**Figure 3:**
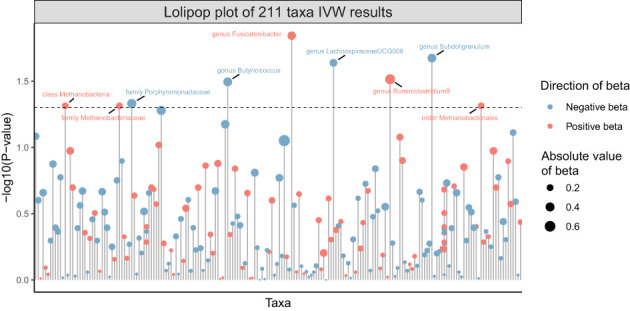
Lollipop plot of 211 Taxa’s analysis results of GM on SS.

**Figure 4:**
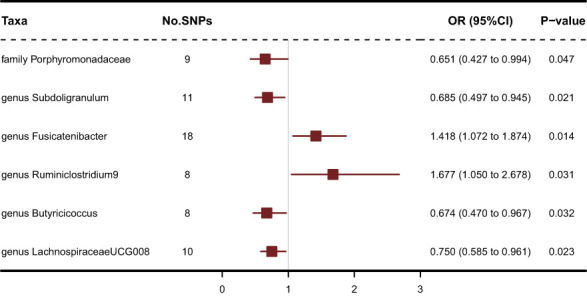
Forest plot of IVW analysis results of the effect of GM on SS.

211 Taxa’s analysis results are shown in the lollipop plot in **Figure 3**. Class Methanobacteria, family Methanobacteriaceae and order Methanobacteriales were excluded from the analysis results because MR-Egger showed a different effect direction than the other four methods (), and the final significant six Taxa forest plots are shown in **Figure 4**. The results of IVW analyses demonstrated that genus Fusicatenibacter (odds ratio (OR) = 1.418, 95% confidence interval (CI), 1.072–1.874, P = 0.0143) and genus Ruminiclostridium9 (OR = 1.677, 95% CI, 1.050–2.678, P = 0.0306) were positively correlated with the risk of SS. Family Porphyromonadaceae (OR = 0.651, 95% CI, 0.427–0.994, P = 0.0466), genus Subdoligranulum (OR = 0.685, 95% CI, 0.497–0.945, P = 0.0211), genus Butyricicoccus (OR = 0.674, 95% CI, 0.470–0.967, P = 0.0319) and genus Lachnospiraceae (OR = 0.750, 95% CI, 0.585–0.961, P = 0.0229) were negatively correlated with SS risk (**Figure 4**). The MR estimates of weighted median indicated that genus Butyricicoccus (OR = 0.611, 95% CI, 0.384–0.972, P = 0.0377) and family Porphyromonadaceae (OR = 0.521, 95% CI, 0.298–0.909, P = 0.0217) served as protective factors for SS (**Supplementary Table S1**). However, none of the results were significant after Bonferroni multiple tests correction (P<0.05/211 = 0.000237). We show significant taxa’s SNPs in **Supplementary Table S2** and **Supplementary Figure S1**. The heterogeneity test revealed no heterogeneity among the individual SNPs. It seemed unlikely that horizontal pleiotropy would distort the impact of the gut microbiota on SS, according to the findings of the MR-Egger regression and MR-PRESSO global test (**Table 1**). Leave-one-out analysis revealed that no one SNP was responsible for the causative estimates of GM and SS, which were displayed in **Supplementary Figure S2**.

**Table 1:** Heterogeneity test, pleiotropy test and MR-PRESSO results of genetic variants.

Taxa	Heterogeneity				Pleiotropy		MR-PRESSO	
	MR Egger		IVW		MR Egger		Global Test	
	Cochran’s Q	P-value	Cochran’s Q	P-value	Egger intercept	P-value	RSSobs	P-value
family Porphyromonadaceae	4.819	0.682	5.311	0.724	0.039	0.506	6.756	0.732
genus Subdoligranulum	5.299	0.808	5.816	0.830	-0.022	0.490	7.116	0.860
genus Fusicatenibacter	14.609	0.553	14.621	0.623	-0.004	0.914	16.421	0.620
genus Ruminiclostridium9	8.863	0.181	8.864	0.263	0.002	0.985	11.699	0.298
genus Butyricicoccus	2.434	0.876	3.132	0.873	0.026	0.436	4.048	0.886
genus Lachnospiraceae UCG008	10.026	0.263	11.081	0.270	0.062	0.386	13.674	0.294

### Mapping SNPs to genes

**Figure 5:**
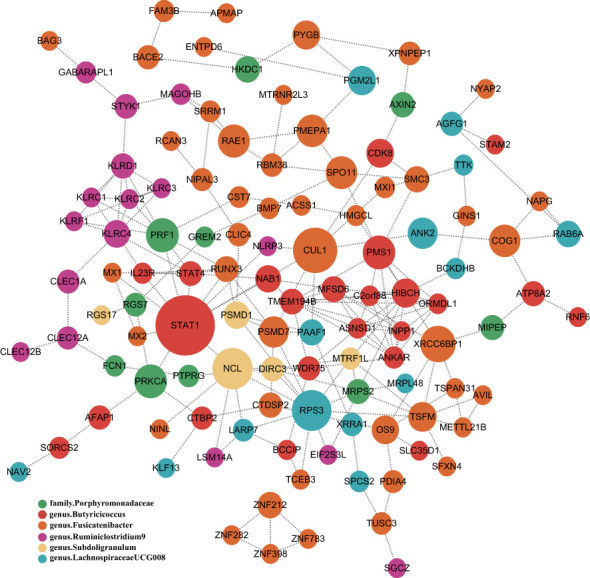
PPI network of six significant GM’s SNPs mapped genes. Genes are represented by circular nodes, and their interactions are represented by edges. The size of the dots represents the Betweenness Centrality score, with high Betweenness Centrality scores being large and low scoring dots being small, and each of the six bacteria-related genes is represented by six different colors.

To obtain more insight into the biological significance of prior findings, we evaluated the functional annotations of the genetic variants used as IVs in MR through FUMA GWAS tool (37). The SNP hit genes are shown in **Supplementary Table S3**. STRING was used to generate PPI networks from 324 significant GM’s SNPs mapped genes, which were then displayed in Cytoscape to predict the interactions and adhesion pathways of common significant GM’s SNPs mapped genes. As shown in the diagram, the PPI network of genes hit by SNPs contains 115 nodes and 202 edges (**Figure 5**).

### Deeper MR analysis of transcriptomic

**Figure 6:**
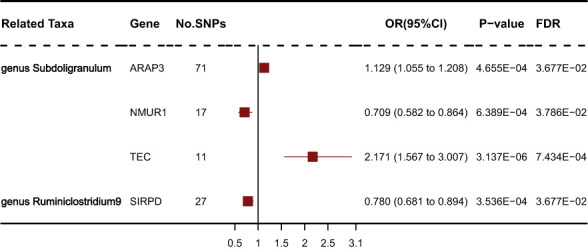
Forest plot of IVW analysis results of the effect of mapped genes on SS.

We obtained gene expression-related SNPs (eQTLs) from the eQTLGen consortium (https://eqtlgen.org/). As illustrated in **Figure 6**, the genetically proxied expression of 4 genes was significantly associated with the risk of schizophrenia at FDR < 0.05. Among these genes, ARAP3, NMUR1, TEC were associated with genus Subdoligranulum. SIRPD was associated with genus Ruminiclostridium9. Other genes were not significantly associated with SS. As illustrated in **Supplementary Table S4**, the effect direction of all five MR approaches was consistent. The heterogeneity test showed no heterogeneity among individual eQTLs. According to the MR-Egger regression results, it seemed implausible that horizontal pleiotropy would bias the effect of gut microbiota on SS (**Supplementary Table S5**).

## Discussion

This was the first study to evaluate the two-way causal link between the GM and SS using a number of complimentary MR methods. This TSMR investigation provided evidence that the presence of family Porphyromonadaceae, genus Subdoligranulum, genus Butyricicoccus, and genus Lachnospiraceae was associated with a lower chance of developing SS, and that genus Fusicatenibacter and genus Ruminiclostridium9 may be factors that increase the likelihood of SS. Further, we performed MR analysis of these genes using these GM-associated SNPs paired on top of the genes, suggesting a causal relationship between these genes and SS, which may indicate that the GM affects SS through these genes.

The immune system and GM interact physiologically during the developing process, and GM has been implicated in the development of chronic inflammatory illnesses and metabolic disorders in humans (7, 41). Recent research suggests that gut dysbiosis affects the immune system in a way that causes autoimmune disorders such rheumatoid arthritis, SLE, systemic sclerosis, ankylosing spondylitis, and Sjögren’s syndrome to develop or worsen (42).

Earlier epidemiological investigations discovered a link between GM and SS. Previous research has shown that when SS patients are compared to healthy persons, the GM’s -diversity is considerably reduced and its -diversity is changed (18, 43, 44). The severity of dry eye symptoms and GM diversity were shown to be correlated (4). The Firmicutes/Bacteroidetes (F/B) ratio is frequently seen to be lowered in individuals with autoimmune diseases (45, 46), which may be a sign of GM dysbiosis (47, 48). Nevertheless, these studies have considerable drawbacks because of variations in ethnicity, gender, or diet between cohorts, limited sample numbers, and various sequencing techniques, which lead to significant variations in the findings among research. As a result, it is difficult to infer the etiology of GM and SS based solely on prior studies.

Molecular mimicry, metabolite alterations, and the collapse of epithelial tolerance are the primary explanations for how the gut microbiota may affect Sjogren’s syndrome (49). One of the most important points is the change of metabolites. Changes in metabolites short-chain fatty acids (SCFAs), which mostly consist of acetic acid, propionic acid, and butyric acid, are one of the significant metabolites produced by gut microbes. SCFAs are essential signaling molecules that control the immune system, cell growth, and metabolism of the host (50, 51). Many SCFA-producing bacteria, including Lachnoclostridium, Lachnospira, and Sutterella, were decreased in systemic autoimmune disorders. It should be noted that these bacteria have a strong pro-regulatory and tolerogenic effect on immunological processes (52). The most frequently cited bacterial product in SS is butyrate. Numerous investigations found that butyrate-producing bacteria were substantially reduced in SS (16, 53, 54), including Faecalibacterium prausnitzii, Bacteroides fragilis, Lachnoclostridium, Roseburia, Lachnospira, and Ruminococcus. This is consistent with our findings. Butyrate, a crucial metabolite produced from the microbiome, supports gut barrier processes by supplying colonic epithelial cells with energy. Furthermore, new studies found that the Clostridia clusters XIVa and IV, as well as butyrate-producing bacteria Bacteroides spp., were crucial for preserving the Treg/Th17 equilibrium. It should be emphasized that the immunomodulatory molecule polysaccharide works in concert with butyrate to support the development of Treg cells. a Bacteroides fragilis descendant (55–57). The mucosa barrier’s capacity to prevent the colonization of harmful microorganisms will be compromised by disruption of the Treg/Th17 balance (16). Basically, butyrate regulates genes associated with the circadian clock to carry out the anti-inflammation function, which has the ability to alter T cell balances and control the frequency of B cells that produce IL-10 and/or IL-17 (58). These various cues point to a possible role for diminished SCFAs or butyrate-producing bacteria in the pathogenesis of SS through altering immune cell frequency or function, mucosal barrier permeability, and possibly salivary gland secretion.

In addition, there is evidence that a bacterial-derived peptide causes clonal expansion of CD8+ T cells, which is associated with autoimmune diseases such as AS and reactive arthritis (ReA) caused by bacterial infections (59), and that GM may act in the same way in SS. HLA-B27 is a member of the HLA Class I family of MHC genes whose role is to present peptide antigens to CD8 T cells. In the feces of individuals with AS, peptide elution experiments have revealed an abundance of bacterial peptides that are identical to known HLA-B27-presented epitopes, indicating a failure in the clearance of these bacteria, and several peptides provided by APCs-B*27+, but not by B27-negative donors, elicited CD8 responses, which is consistent with these peptides activating the adaptive immune system in AS (60). Within the setting of innate immune activation, proliferation of self-reactive Th17 cells, or microbial mimicry, the initial infectious stage is followed by hyperactivation and disturbed self-tolerance (61). Therefore, CD8 T cells are kept in a state of high activation and do not experience senescence, which results in less effective responses to foreign antigens, possibly as a result of ongoing exposure to bacterial adjuvant (62, 63). This also affects immunological priming and increases the generation of proinflammatory cytokines (TNF, IL-23) that contribute to the clinical signs of joint and gastrointestinal inflammation that are frequently present in AS (60, 64–66). The above studies on GM and AS further demonstrate the causal relationship we found between GM and SS, which is also an autoimmune disease, suggesting that GM may contribute to autoimmune disease through a common pathway. To further explore the immune mechanisms behind the causal relationship between GM and SS, we also built a PPI network using six significant GM’s SNPs mapped genes, based on an in-depth understanding of protein biology and prediction of drug targets.

Among the GM-related genes that were causally associated with SS, TEC gene encodes a non-receptor protein tyrosine kinase with a pleckstrin homology domain that is involved in the intracellular signaling mechanisms of cytokine receptors, lymphocyte surface antigens, heterotrimeric G protein-coupled receptors, and integrin molecules, key players in the regulation of immune functions, an integral component of T cell signaling, and plays a distinct role in T cell activation (67, 68). TEC may be associated with possible pathogenic variants of autoimmune diseases such as SS by regulating T cell activation and T cell receptor signaling pathways (69). This may suggest that GM influences the occurrence of SS through TEC. Other genes in this study have not been previously reported to be related to SS, which may be a new finding and provide clues for future studies on the mechanism of action between GM and SS

This study has a number of advantages. By removing confounding variables and reversing the causal inference process, MR analysis was used to establish the causal relationship between gut microbiota and SS. The most comprehensive GWAS meta-analysis was used to acquire genetic variations of the gut microbiome, guaranteeing the reliability of the analytical tools. The MR-PRESSO and MR-Egger regression intercept term analyses were used to identify and rule out horizontal pleiotropy. In addition, a network-based approach is developed to investigate the gene expression patterns from 6 significant GM’s SNPs mapped genes and identified molecular targets that may help as potential biomarkers of GM’s SNPs mapped genes in this study. It could also provide crucial information about their effects on SS.

This research does have some limitations, though. First, the study data refer only to people of European ancestry and do not include people from other regions. It is still unclear, therefore, whether the results can be regarded as representative of the total community. Second, although it is challenging to determine the extent of sample overlap, there was probably some overlap between the exposure and result research participants. Fortunately, the robust methods employed in this investigation (F statistic substantially greater than 10) should minimize any potential bias brought on by sample overlap (70). Third, the variability of the MiBioGen meta-analysis was extremely high, with only 9 taxa identified in> 95% of the samples, which may have affected the accuracy of the results of this study. Fourth, with only 2247 SS cases in the FinnGen GWAS data, this small sample size may not produce a sufficiently valid beta value, resulting in less statistical power. However, the FinnGen SS GWAS still provides valuable insights into genetic architecture and can serve as a useful starting point for further investigations with larger sample sizes or complementary approaches. Fifth, we did not consider gender factors in the analysis of data, and whether gender differences have an impact on the results needs further study due to data unavailability. Sixth, we did not find GWAS data on “dry eyes” and “dry mouth” for further differentiation studies. Finally, the results were not significant after multiple corrections were performed. But adopting a strict multiple testing correction would have likely been unduly cautious given the biological plausibility and the multi-stage statistical approach, which may have overlooked possible strains that are causally associated to SS. In addition, it is essential to interpret the results of Mendelian randomization analyses in concert with additional measures such as instrument strength and sensitivity analyses as we have done in this study. As a result, we remain optimistic that our research has academic implications. Future studies should aim to validate our current findings with larger sample sizes and diverse populations to establish the robustness of the results obtained. In addition, it would be worthwhile to carry out replication analyses using different MR methods to confirm the identified genetic associations. Meanwhile, the search for mediating variables in the causal chain of GM and SS is also important for the prevention and treatment of SS.

## Conclusions

In summary, we thoroughly evaluated the probable causal relationship between the gut microbiota and SS. Four other bacterial traits had a negative causative direction with SS, whereas two further bacterial features displayed a positive causal direction. Many intestinal bacterial species discovered in this study that may have decreased the incidence of SS may hold promise for SS prevention and therapy.

## Data availability statement

Publicly available datasets were analyzed in this study. This data can be found here: Gut microbiota GWAS data is from MiBioGen(https://mibiogen.gcc.rug.nl/). Sjögren’s Syndrome GWAS data is from FinnGen R8 release(https://www.finngen.fi/en).

## Author contributions

YC and MZ conceived the presented idea. HL and YC performed the manuscript writing. YC and WX was involved in acquisition and processing of data. HL was involved in interpretation of data. YC and HL have contributed equally to this work and share first authorship. All authors contributed to the article and approved the submitted version.
