# Supplementary material for: Gut microbiota and Sjögren’s syndrome: a two-sample Mendelian randomization study
Source: Front Immunol. 2023 Jun 13;14:1187906. doi: 10.3389/fimmu.2023.1187906 (PMC10299808; doi:10.3389/fimmu.2023.1187906)
Supplement: Supplementary file 1 [file Image_1.pdf]

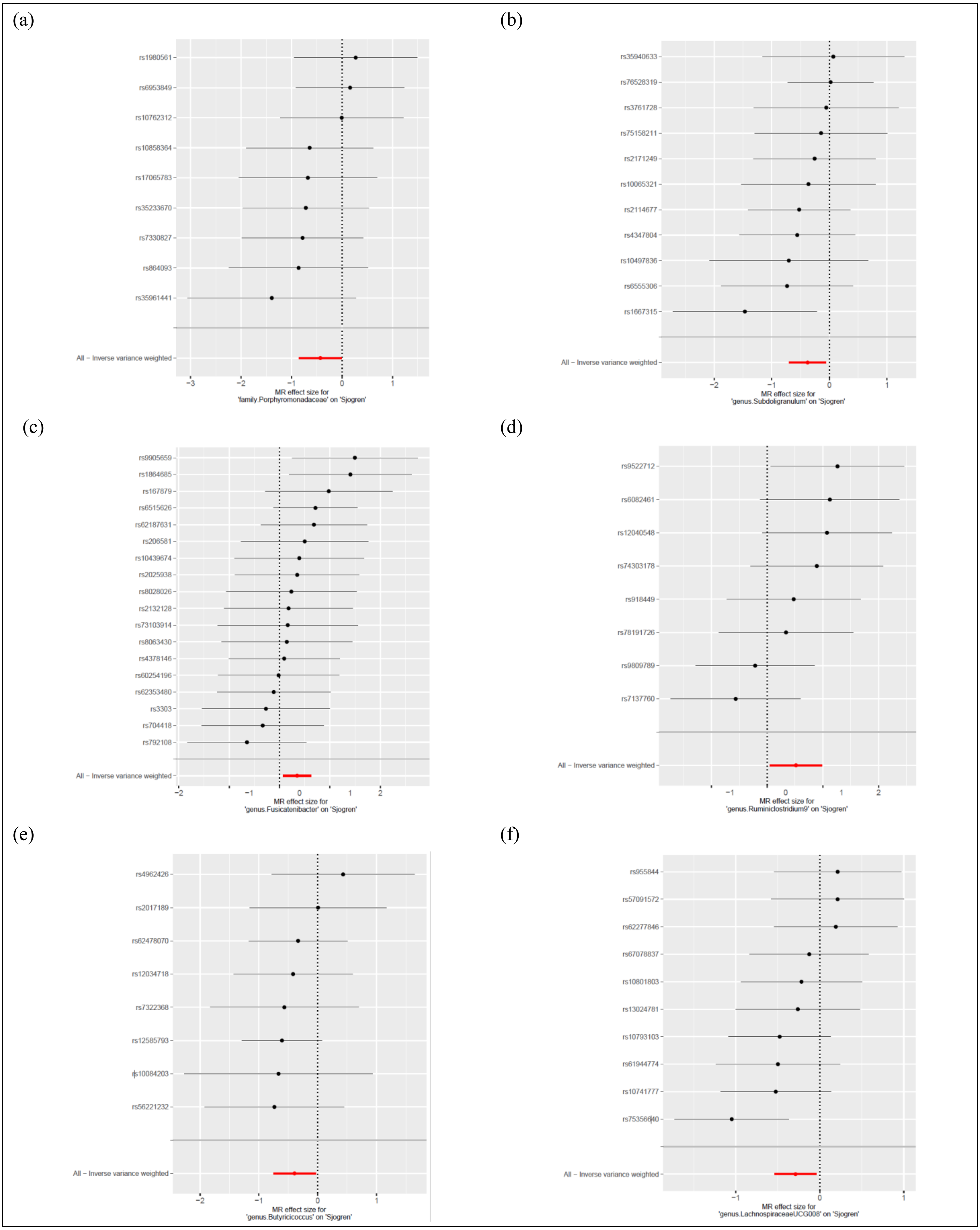

Figure S1 (a).SNP level forest plot for family Porphyromonadaceae on Sjogren's syndrome. (b). SNP level forest plot for genus Subdoligranulum on Sjogren's syndrome. (c).SNP level forest plot for genus Fusicatenibacter on Sjogren's syndrome. (d).SNP level forest plot for genus Ruminiclostridium9 on Sjogren's syndrome. (e).SNP level forest plot for genus Butyricicoccus on Sjogren's syndrome. (f).SNP level forest plot for genus LachnospiraceaeUCG008 on Sjogren's syndrome.

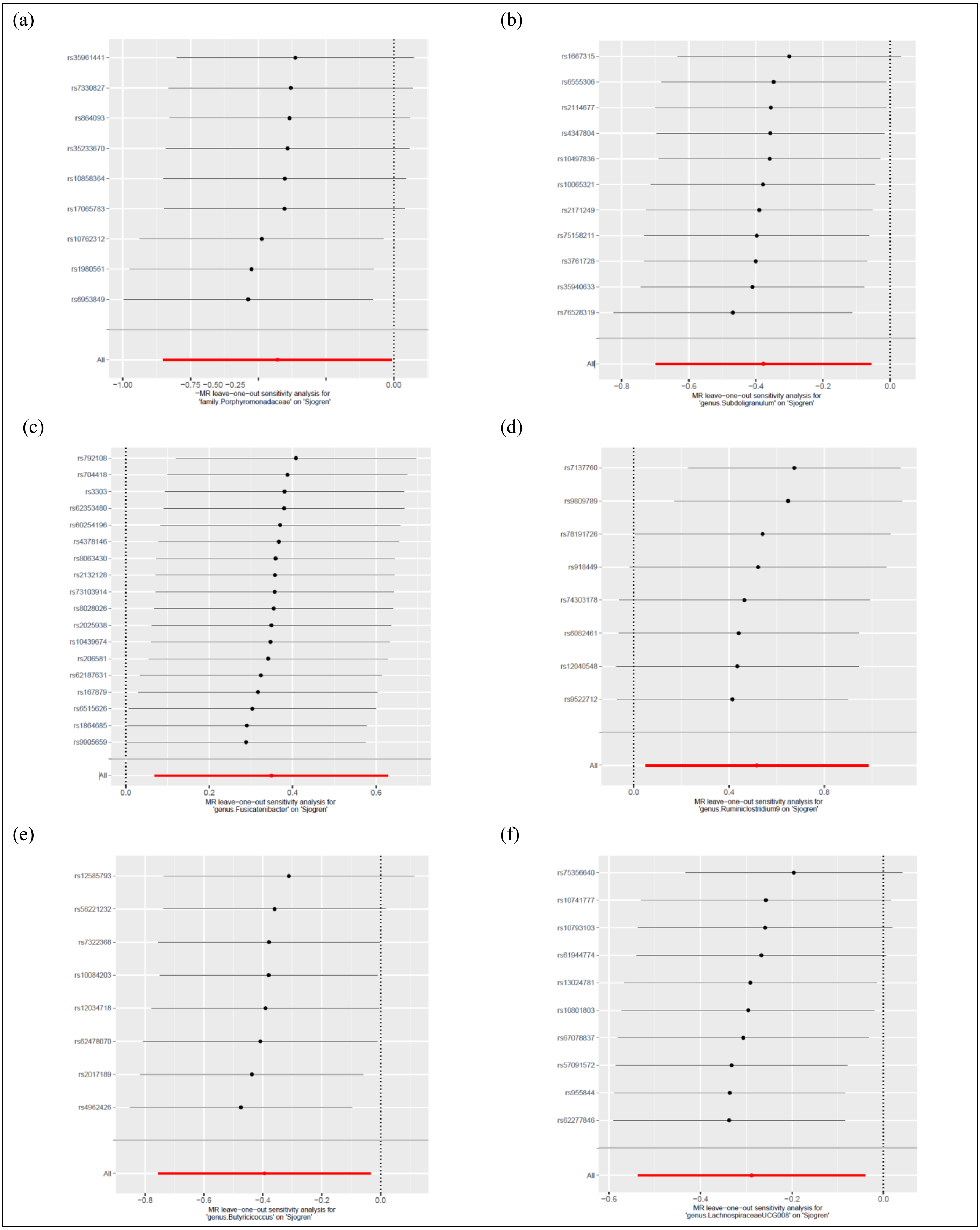

Figure S2 (a). Leave-one-out plot for family Porphyromonadaceae on Sjogren's syndrome. (b). Leave-one-out plot for genus Subdoligranulum on Sjogren's syndrome. (c). Leave-one-out plot for genus Fusicatenibacter on Sjogren's syndrome. (d). Leave-one-out plot for genus Ruminiclostridium9 on Sjogren's syndrome. (e). Leave-one-out plot for genus Butyricicoccus on Sjogren's syndrome. (f). Leave-one-out plot for genus LachnospiraceaeUCG008 on Sjogren's syndrome.
